# Three pillars for digital health assessment in LMICs: a mini review of technology benchmarking, digital health literacy, and social media listening

**DOI:** 10.3389/fpubh.2026.1790605

**Published:** 2026-09-29

**Authors:** Anggi Septia Irawan, Bence Döbrössy, Edmond Girasek

**Affiliations:** 1 Institute of Behavioural Sciences, Semmelweis University, Budapest, Hungary; 2 Department of Psychology and Healthcare Management, Faculty of Health and Sport Sciences, Széchenyi István University, Győr, Hungary

**Keywords:** digital health, digital health literacy, health technology assessment, mHealth, social media listening, technology benchmarking

## Abstract

Digital health has emerged as a critical component of healthcare delivery in low- and middle-income countries (LMICs), offering opportunities to strengthen access, monitoring, and engagement across diverse populations. However, existing evaluations often focus narrowly on technological functionality while overlooking broader contextual factors such as digital literacy and public discourse. This mini-review proposes an integrative framework for digital health assessment built around three complementary pillars: technology benchmarking, digital health literacy, and social media listening. A structured literature search of PubMed, supplemented by Web of Science, was conducted to identify representative studies published between 2020 and 2026 relevant to digital health implementation in LMICs. Fifteen studies were selected to provide illustrative evidence across the three domains. The proposed framework highlights the need for multidimensional evaluation approaches that move beyond conventional app-based metrics. While broadly applicable across healthcare sectors, the framework offers particular relevance for population groups requiring sustained engagement, including maternal, child, and adolescent health contexts. This review contributes a practical conceptual model for researchers, policymakers, and developers seeking to strengthen digital health strategies in resource-constrained settings.

## Introduction

Digital health encompasses telemedicine, mobile health (mHealth), artificial intelligence-assisted diagnostics, electronic health records, and wearable devices. These technologies can make healthcare delivery faster, more efficient, inclusive, and accessible, particularly in low- and middle-income countries (LMICs), where infrastructure, financial constraints, and educational inequities continue to limit access (1). In pediatric care, timely interventions are essential; mHealth apps, SMS reminders, and social media communication can help connect healthcare providers with caregivers. Their effectiveness must nevertheless be evaluated critically to ensure that they deliver meaningful benefits (2).

Digital literacy is a crucial aspect of human capacity in the digital era, particularly when individuals engage as users of digital health technologies. It not only influences how people access, interpret, and evaluate health information but also determines their ability to effectively use digital tools for health management and decision-making. Therefore, assessing digital literacy should be considered an essential component of any digital health evaluation, as it directly affects the accuracy, usability, and overall impact of such technologies. Studies have shown that higher levels of digital literacy are associated with better health outcomes and greater engagement with digital health platforms (3, 4).

The COVID-19 pandemic further accelerated digital health uptake, driving the widespread adoption of telemedicine platforms, mobile applications, and digital surveillance tools to sustain essential services while reducing infection risk (5). These interventions offer scalable and cost-effective solutions to longstanding healthcare barriers, ranging from nutrition education, maternal and child health to chronic disease monitoring and public health reporting (4).

Beyond bridging access gaps, digital health supports healthcare workforce capacity building and improves patient-provider partnerships by facilitating decision-making processes, remote consultations, and personalized interventions (6). Innovative projects, such as EU-funded initiatives for training health professionals in digital competencies, underscore the importance of building both digital infrastructure and human capacity (7). Accordingly, this mini-review had three objectives: (1) to propose an integrated framework for digital health assessment in LMICs based on technology benchmarking, digital health literacy, and social media listening; (2) to synthesize illustrative evidence showing how each pillar contributes to the evaluation of digital health interventions; and (3) to examine how the three pillars can be combined to support context-sensitive design, implementation, and policy decisions.

## Methods

This mini-review conducted a structured PubMed search in September 2025, updated in March 2026, and a supplementary Web of Science search to broaden coverage of interdisciplinary digital-health and information-technology literature. Studies published between 2020 and 2026 were screened for relevance to the three conceptual pillars. The PubMed search returned 312 records; the supplementary Web of Science search contributed one additional record. After screening and eligibility assessment, 15 studies were selected for illustrative narrative synthesis rather than exhaustive effect estimation.

*(“digital health” OR “mHealth” OR “eHealth” OR “telehealth” OR* “mobile health application”*)*

*AND*

*(“evaluation” OR “assessment” OR “technology benchmarking” OR “usability assessment” OR “implementation evaluation” OR* “digital health literacy” *OR “eHealth literacy” OR* “social media analysis” *OR “infodemic management”)*

*AND*

*(*“low- and middle-income countries” *OR “LMICs” OR “resource-limited settings” OR “developing countries” OR “global south”)*

PubMed was selected as the primary database because of its strong coverage of biomedical, public-health, and implementation research. Web of Science was added in response to the interdisciplinary nature of the framework and to capture relevant technology and information-science sources that may not be represented adequately in PubMed. The same date limits, conceptual domains, and LMIC relevance criteria were applied across both sources. The search remained purposive and appropriate to a mini-review: eligible studies had to address at least one of the three pillars or provide cross-cutting evidence that linked two or more pillars. Cross-cutting evidence was treated as a supporting analytic category, not as a fourth pillar.

Study selection was purposive rather than random. Titles and abstracts were screened first, followed by full-text assessment. Studies were included when they: (1) were peer-reviewed empirical studies, reviews, or substantive implementation analyses published in English between 2020 and 2026; (2) examined digital health technologies, digital health literacy, social media data, or digital-health implementation and evaluation; (3) addressed an LMIC setting or provided a clearly transferable framework relevant to resource-constrained health systems; and (4) contributed evidence to at least one of the three predefined pillars. Studies were excluded when they fell outside the publication period, were unrelated to health, did not contain evaluative or implementation-relevant evidence, focused exclusively on high-income settings without clear LMIC transferability, were editorials or commentaries without substantive evidence, duplicated another record, or lacked sufficient methodological detail.

The three pillars were defined *a priori* from the conceptual aim of the review and from recurring gaps identified in the digital-health evaluation literature: technical quality, users’ capacity to engage with digital systems, and the surrounding public-information environment. Eligible studies were therefore mapped deductively to the pillar that best represented their primary contribution. During narrative synthesis, a cross-cutting evidence category was developed inductively for studies that linked two or more pillars. This category supports the integration of the framework and does not constitute a fourth pillar.

## Results

The mini-review included 15 studies (Figure 1) published between 2020 and 2026. Evidence was mapped to three conceptual pillars. A separate cross-cutting category was retained only for studies that connected two or more pillars; it is not an additional pillar:

1. Technology Benchmarking (*n* = 4) – focused on benchmarking diagnostics, registries, infrastructure assessment, and health technology assessment (HTA) frameworks.
2. Digital Health Literacy (*n* = 4) – included interventions for low-literacy populations, scoping reviews of digital literacy determinants, and frameworks for workforce competency.
3. Social Media Listening (*n* = 3) – focused on infodemic monitoring, big data surveillance, and methodological insights for using social media platforms for public health.
4. Cross-Cutting Evidence (*n* = 4) – studies spanning two or more pillars and providing integrative evidence on implementation, classification, or health-system adoption. This is an evidence-mapping category rather than a fourth assessment pillar.

**Figure 1:**
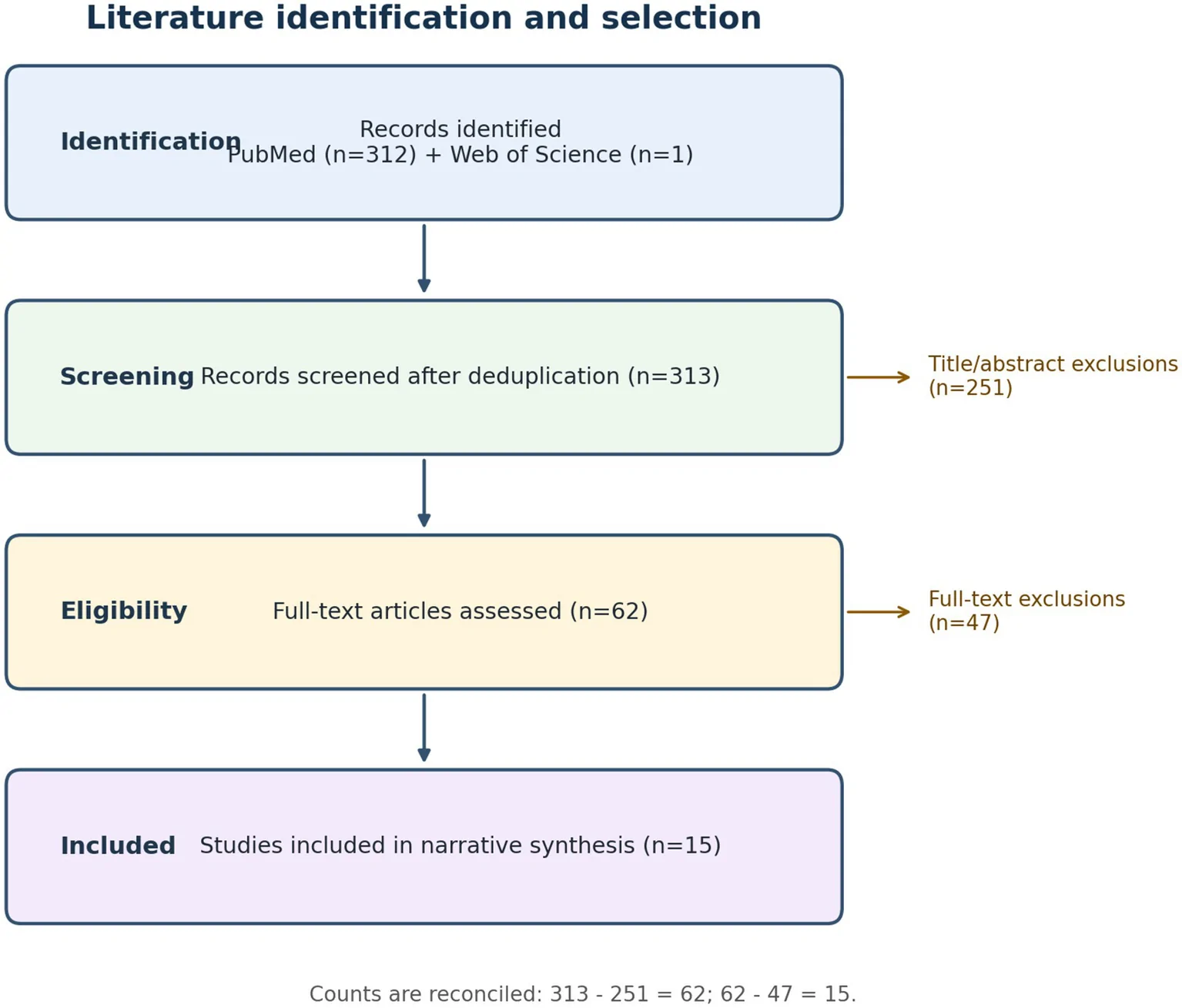
Literature identification and selection process across PubMed and Web of Science.

These studies represented a growing emphasis on holistic assessment approaches in LMICs, integrating infrastructure benchmarking, end-user literacy evaluation, and population-level surveillance methods (Table 1).

**Table 1:** Illustrative studies informing the three-pillar framework.

Study (Year)	Country/region	Primary pillar	Secondary relevance	Study focus	Contribution to framework
Yadav et al. (8)	10 LMICs	Technology benchmarking	—	Diagnostic service benchmarking across LMIC facilities	Provides measurable indicators for service availability and performance benchmarking
Waqar et al. (9)	Resource-limited settings	Technology benchmarking	Systems strengthening	Perioperative registry evaluation	Demonstrates registry-based benchmarking for surgical quality improvement
Patel et al. (10)	India	Technology benchmarking	Policy implementation	District-level infrastructure assessment	Identifies barriers and scalable models for digital health evaluation
Leckenby et al. (11)	Global/transferable to LMICs	Technology benchmarking	Regulatory systems	Sandbox and HTA frameworks	Offers adaptable benchmarking frameworks for digital health technologies
Salim et al. (12)	LMIC-relevant populations	Digital health literacy	User-centered design	App feasibility for low-literacy adults	Highlights inclusive design for literacy-constrained users
Choukou et al. (13)	Vulnerable populations	Digital health literacy	Social media dynamics	Interventions during COVID-19	Demonstrates literacy-based responses to misinformation
Nazeha et al. (14)	Global/workforce settings	Digital health literacy	Capacity building	Digital competency review	Establishes provider literacy as a prerequisite for adoption
Rachmani et al. (15)	Indonesia	Digital health literacy	Technology adoption	Readiness for the SatuSehat national health app	Shows how digital and health literacy inform usability and adoption in an LMIC setting
Van Heerden et al. (16)	Sub-Saharan Africa	Social media listening	Public health surveillance	Social media analytics for HIV surveillance	Demonstrates surveillance potential in LMIC public health systems
Blandi et al. (17)	Global/applicable to LMICs	Social media listening	Information ecosystems	Infodemic and online behavior analysis	Characterizes misinformation and trust dynamics
Seiler et al. (18)	LMICs	Social media listening	Behavior change	Systematic review of social media interventions	Identifies evaluation, engagement, theory, and privacy gaps in LMIC interventions
Till et al. (19)	Sub-Saharan Africa	Cross-cutting systems insight	All three pillars	Maternal and child health digital tools	Maps digital health typologies relevant to LMIC health systems
Kosowicz et al. (20)	Vietnam	Cross-cutting systems insight	Technology benchmarking	NICE-tier digital classification	Supports structured adoption pathways
Yew et al. (21)	LMIC hospital settings	Cross-cutting systems insight	Systems implementation	Post-COVID barriers and enablers	Provides system-level adoption insights
Mishra et al. (22)	Cambodia	Cross-cutting systems insight	User-centered design	Implementing app for Acute fever management	Provides system-level adoption insights

These categories were derived based on recurring themes and methodological approaches identified across the studies. Each category represents a distinct dimension of the findings, allowing for a more structured interpretation and comparison. The categories are explained in detail below.

### Technology benchmarking

Four studies explored approaches to benchmarking digital health tools and infrastructure in LMICs (8–11). Yadav quantified diagnostic availability across 10 LMICs using standardized Service Provision Assessment indicators, providing crucial data for policy and planning (8). Waqar highlighted the value of perioperative registries as tools for benchmarking surgical quality, demonstrating their potential to improve outcomes in resource-limited settings (9). Patel introduced a district-level model in India to identify barriers to scaling digital infrastructure, offering methods that could be applied to other LMICs (10). Finally, Leckenby proposed a “sandbox approach” for regulatory and health technology assessment innovation, aimed at evaluating emerging digital health technologies (11). These studies collectively contribute to efforts in establishing benchmarks for digital health infrastructure in LMICs. Each approach provides essential insights for enhancing the effectiveness of digital health tools in improving healthcare quality and access in resource-constrained settings.

### Digital health literacy

Four studies investigated digital health literacy (DHL) among vulnerable populations, health workers, and digital-health users (12–15). Salim et al. showed the feasibility of a self-management app designed for adults with limited health literacy (12). Choukou et al. emphasized the role of digital literacy in countering COVID-19 infodemics among vulnerable groups (13). Nazeha et al. synthesized digital competency frameworks for health professionals (14). The supplementary Web of Science search identified Rachmani et al.’s Indonesian study of readiness to use the SatuSehat national health app, illustrating how health literacy and digital health literacy shape usability and adoption in an LMIC context (15).

### Social media listening

Social media platforms have emerged as valuable tools for surveillance, communication, and intervention in LMICs. Three studies addressed social data and public-health applications (16–18). Van Heerden et al. highlighted social media as a potential HIV surveillance tool in sub-Saharan Africa (16). Blandi et al. examined infodemic trends and online information-seeking during COVID-19 (17). Seiler et al.’s systematic review of social media-based health-behavior interventions in LMICs identified important gaps in engagement measurement, theoretical grounding, evaluation, and privacy reporting (18).

### Cross-cutting reviews and landscape analyses

Four cross-cutting studies synthesized lessons on digital health integration, barriers, and classification frameworks (19–22). Till Bergman’s study offered typologies for digital health technologies specifically in maternal and child health within LMIC health systems, providing valuable insights for their adoption (19). Kosowicz introduced a NICE-tier classification for digital health technologies in Vietnam, offering a practical framework to guide their implementation and benchmarking (20). Additionally, these reviews collectively provide key frameworks and typologies, such as the NICE tiers, to support the integration and scaling of digital health tools in resource-constrained settings and App in Cambodia for acute fever management (21, 23). These works contribute significantly to understanding the barriers and challenges in adopting digital health technologies, while offering practical guidance on their classification and integration in LMICs.

## Discussion

The findings indicate that no single assessment approach can sufficiently explain whether a digital health intervention is technically robust, understandable and usable, and responsive to the information environment in which it operates. Therefore, Figure 2 illustrates the proposed relationship among the three pillars, demonstrating how their combined application supports iterative assessment and continuous improvement.

**Figure 2:**
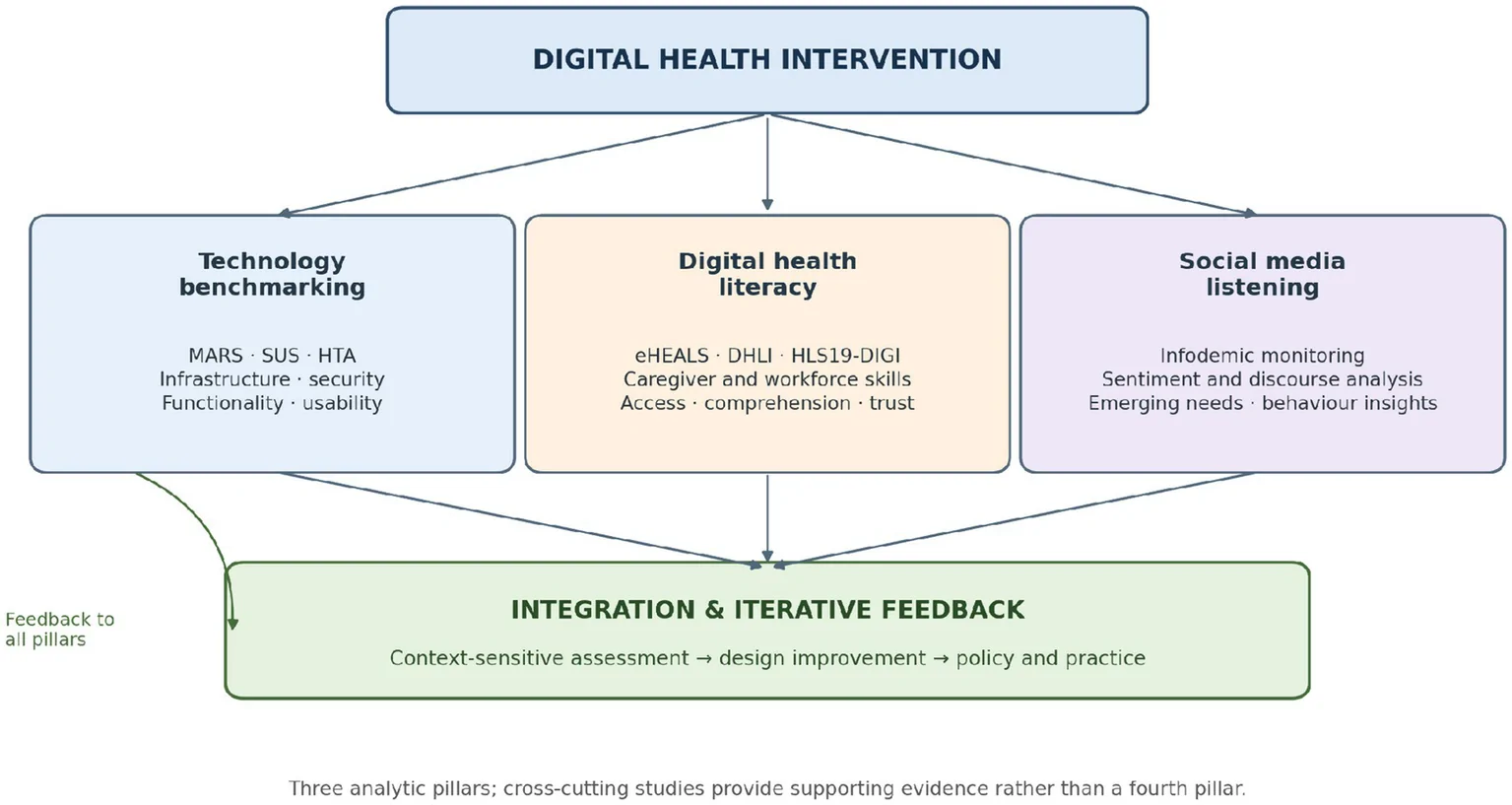
Integrated three-pillar framework for digital health assessment. Cross-cutting evidence supports integration but does not constitute a fourth pillar. DHLI, Digital Health Literacy Instrument; eHEALS, eHealth Literacy Scale; HLS19-DIGI, Health Literacy Survey 2019 Digital Health Literacy instrument; HTA, Health Technology Assessment; MARS, Mobile App Rating Scale; SUS, System Usability Scale. (SUS, System Usability Scale; HTA, Health Technology Assessment).

### Technology benchmarking

Technology benchmarking, more broadly, entails the systematic evaluation of digital health applications using standardized tools (see Figure 2). For instance, the Mobile App Rating Scale (MARS) provides a multidimensional assessment of engagement, functionality, esthetics, and information quality (24). MARS has been validated as a reliable tool for evaluating health apps in various domains and is particularly useful for assessing user-centered design in LMICs settings (25).

Recently, we conducted a benchmarking study of pediatric-focused digital health applications available in the Indonesian App Store. MARS tools revealed that while the apps generally scored high in functionality and esthetic design, engagement, and information quality varied considerably. Only 33% of the apps incorporated personalized guidance and feedback features, and fewer than half offered community-interaction tools. These deficits are particularly detrimental in rural settings, where offline functionality, linguistic localization, and interactivity are essential for uptake and sustainability (26).

Benchmarking highlights how these apps often fall short in terms of user engagement and interactivity, which are crucial for long-term behavior change (27). The use of usability scales, such as the System Usability Scale (SUS) and User Experience Questionnaire (UEQ), can complement the MARS to provide holistic insights (28). For instance, some apps score relatively high in terms of content relevance but lack customizable features for rural users with intermittent Internet access. Other benchmarking approaches include heuristic evaluations (29), ISO/IEC 25010 software quality standards, and app store review analyses (30). Integration with electronic medical records (EMRs) and data security measures also forms part of benchmarking in contexts where personal data protection laws may be weak or inconsistently applied (31).

### Digital health literacy

The importance of digital health literacy (DHL) is increasingly recognized, particularly among vulnerable populations and health workers. In one survey, nearly half of the individuals who had downloaded an mHealth app reported discontinuing it, with the majority citing a high burden of data entry or confusion with app usage (32). Studies in LMICs have shown that low DHL correlated with poor health-seeking behavior, delayed treatment, and misuse of health apps (33, 34). Tools such as the eHealth Literacy Scale (eHEALS) and Digital Health Literacy Instrument (DHLI) offer frameworks for measuring these competencies (see Figure 2) (35).

Addressing DHL gaps requires context-specific training, community-based digital education programs, and the inclusion of local languages and dialects in app content (36). Gender dynamics also affect access; women, who are often primary caregivers, may lack smartphone access or digital training because of patriarchal norms (37). One reliable instrument for measuring digital health literacy is the HLS19-DIGI. The HLS19-DIGI scale was sufficiently unidimensional, and no differential item functioning or disordered response categories were observed (38).

Digital health literacy (DHL) has emerged as a critical subdomain of health literacy, particularly in the era of rapidly expanding digital health technologies. It is broadly defined as the ability to locate, comprehend, critically assess, and apply health information obtained from digital platforms to make informed decisions. This competency is essential not only for navigating digital health tools but also for fostering effective health-related decision-making among individuals and communities (39).

In low- and middle-income countries, including rural regions, digital health literacy is pivotal in determining the success of mobile health (mHealth) initiatives. Given the uneven distribution of technological infrastructure and educational attainment, disparities in DHL among parents and caregivers can significantly hinder the adoption and sustained use of digital health tools by these groups. For example, one user experience study revealed that nearly 50% of individuals who had initially downloaded an mHealth application discontinued its use. The most frequently cited reasons included the complexity of navigation, high data input burden, and confusion regarding the app’s functions (32).

Empirical studies in LMICs settings have consistently demonstrated that limited DHL is associated with suboptimal health-seeking behaviors, delayed treatment initiation, and inappropriate use of digital health resources. Such limitations can compromise the effectiveness of mHealth interventions designed to support maternal and child health, nutrition, and chronic disease management (34).

Given these challenges, accurately measuring individuals’ digital health literacy is essential to identify gaps and guide effective interventions. Various validated measurement instruments have been developed to systematically assess DHL. The *eHealth Literacy Scale* (eHEALS) remains one of the most widely used tools for measuring individuals’ perceived skills in finding and evaluating online health information (3). Recently, the *Digital Health Literacy Instrument* (DHLI) was introduced, offering a multidimensional approach that includes operational skills, navigation, evaluation, and privacy management (35). In addition, the *HLS19-DIGI*, a digital extension of the Health Literacy Survey 2019, has proven to be a reliable and unidimensional instrument for measuring DHL across populations. It showed no significant issues with differential item functioning or response category disorders, making it suitable for comparative and cross-national research (38).

Addressing the gaps in DHL requires culturally and contextually sensitive strategies. Community-based digital education programs, especially those that incorporate local languages and dialects, are vital for ensuring inclusivity and comprehensibility. Furthermore, attention must be paid to gender-based disparities in digital access and use. In many patriarchal societies, including rural Indonesia, women who often serve as the primary caregivers may face limited access to smartphones or lack digital training, further exacerbating health inequalities (40).

### Social media listening

This study seeks to introduce the value of social media listening in healthcare and propose its greater integration, particularly in contexts with high digital penetration but limited traditional health infrastructure (41). In many countries, healthcare providers have not yet fully embraced social media as a legitimate data source. A national survey conducted in the United States found that nearly half of the physicians and medical students expressed skepticism about the effectiveness of online communication in improving patient–physician relationships. This indicates a substantial trust gap and highlights the need for improved digital literacy among healthcare professionals (42).

However, social media platforms offer a rich environment for understanding the evolving relationship between healthcare systems and societies. Online interactions through comments, shares, or discussions mirror real-world sentiments and provide a public space where patients, caregivers, and providers co-create narratives about health and wellness. These interactions also illustrate the concept of partnership in digital health, where the traditional roles of patients and providers become more interactive and horizontal (43).

Social listening is particularly relevant in LMICs, where conventional health surveillance systems may be underdeveloped. In such contexts, social media offers an opportunity to understand community experiences, identify emerging health concerns and improve service delivery. For instance, discussions around stunting, caused by chronic malnutrition, can provide health professionals with valuable qualitative data on public knowledge gaps, misinformation, or systemic barriers to nutrition (44).

Technological tools for social media scraping and monitoring have evolved significantly, enabling large-scale data extractions and sentiment analyses. Platforms such as Brandwatch, Talkwalker, and NetBase are primarily used in the private sector; however, academic researchers and policymakers have only recently begun exploring their application in public health. Unlike election monitoring, where social media has become a critical factor, healthcare still lags in adopting such intelligence systems (45, 46). In countries such as Indonesia, where mobile and social media usage is high, social listening can serve as a crucial tool for improving maternal and child health by identifying misinformation and gaging public engagement with health topics (47).

Numerous examples from high-income countries highlighted the successful use of social media in health care. For instance, the Mayo Clinic established its Social Media Center to train healthcare professionals in digital engagement and share evidence-based health information with the public (41). Similarly, the Centers for Disease Control and Prevention (CDC) has used platforms such as Twitter and Facebook to run large-scale public health campaigns, particularly during health emergencies (48). The Cleveland Clinic’s active social media presence, including interactive Q&A sessions and educational videos, has helped increase patient access to trusted medical information (49).

Additionally, online communities have emerged as vital sources of peer support, especially for individuals with chronic conditions such as diabetes and cancer. These platforms allow users to exchange experiences, reduce isolation, and improve their self-management. The #ThisIsPublicHealth campaign by the Association of Schools and Programs of Public Health (ASPPH) exemplifies how social media can promote health awareness and education through community-driven storytelling and visual engagement (50).

### Cross-cutting reviews and landscape analyses

Despite the widespread availability of digital communication tools and social media in both developing and developed countries, their adoption in clinical settings remains limited due to skepticism. For example, physicians at an academic medical center in Lebanon expressed reluctance to use online applications and social media platforms to communicate with patients. This hesitation highlighted the challenges of integrating virtual communication into healthcare, emphasizing the need to assess healthcare providers’ perceptions and develop supportive policies. Rather than resisting this new communication paradigm, healthcare institutions should proactively embrace digital tools to enhance patient care and engagement (51).

Digital surveillance presents diverse opportunities for monitoring disease trends and detecting outbreaks in near real-time. These techniques include augmenting traditional data sources, mapping geographic spread, and optimizing existing surveillance frameworks. For instance, Google search data have been successfully integrated as a “virtual provider” to enhance influenza-like illness surveillance, supplementing outpatient reports. Similarly, Twitter data have helped identify restaurants that are potentially linked to foodborne outbreaks, facilitating targeted inspections. In Madagascar, Google search trends were used alongside healthcare statistics to forecast plague outbreaks, and geolocation data from Twitter combined with air traffic patterns traced the spread of the Chikungunya virus (52).

Beyond infectious disease monitoring, digital data from social media, online surveys, and mobile applications provide critical insights into public health factors, such as dietary habits, healthcare access, and socioeconomic conditions influencing nutrition and child growth. Geospatial analysis of satellite imagery and mobile phone usage helps identify patterns of vector-borne diseases, thereby supporting precise interventions and resource distribution. Integrating mobile health applications into surveillance enables real-time data collection on children’s growth and nutrition, facilitating early detection and timely intervention. This digital ecosystem empowers health officials to identify the underlying factors driving stunting and tailor public health responses (52).

Digital health interventions encompass a broad spectrum of technologies, mobile applications, telemedicine platforms, and web-based educational resources that are increasingly employed to promote maternal and child health in resource-limited settings. These technologies offer innovative solutions to overcome barriers, such as restricted access to healthcare facilities and knowledge gaps among caregivers. In stunting prevention, digital tools address various aspects of child development, including nutrition, immunization, and early stimulation. For example, nutrition-focused mobile applications provide parents with personalized feeding guidance, growth tracking, and reminders for vaccinations and health check-ups. Telemedicine connects families with pediatricians and nutritionists for remote consultations, and interactive online modules educate caregivers about breastfeeding, complementary feeding, and hygiene practices. These digital resources empower parents with evidence-based knowledge that is crucial for optimizing child nutrition and health outcomes (19).

Additional work by Aboye and colleagues provides important contextual support from sub-Saharan Africa. Their comparative review found that mHealth use in the region relies heavily on SMS- and call-based approaches and frequently remains at pilot or small-scale implementation stages, despite its relevance to maternal, child, infectious-disease, and chronic-care needs (53). Their qualitative study in Ethiopia further identified interconnected barriers and enablers across patients, physicians, and health-system executives, including language and awareness, users’ mHealth capabilities, interoperability, competing system priorities, mobile access, perceived usefulness, and the need for user-centered design (54). These findings reinforce the proposed framework: infrastructure and interoperability require technology benchmarking, language and user capabilities require digital health literacy assessment, and context-specific perceptions and trust require continuous attention to the wider information environment.

### Three pillars for digital health assessment

A comprehensive framework for evaluating digital health interventions integrates technology benchmarking, digital health literacy (DHL), and social media listening (SML). This triadic model is especially pertinent to pediatric care in low- and middle-income countries (LMICs), where infrastructural, educational, and cultural barriers complicate health communication.

- Technology benchmarking assesses the technical quality of digital health applications using standardized tools such as the Mobile App Rating Scale (MARS), System Usability Scale (SUS), and heuristic evaluations. However, these metrics often overlook the user context.
- Digital health literacy research bridges this gap by exploring why some users discontinue the use of an app despite favorable technical ratings. Issues such as comprehension difficulties, perceived irrelevance, and distrust are common barriers to using eHealth tools (55).
- Social media listening captures emergent narratives and public sentiments that shape health behaviors, sometimes more powerfully than formal education (49).

Isolated interventions risk ineffectiveness if they ignore this complexity of the problem. For example, Indonesia’s digital stunting reduction programs may exclude vulnerable communities when implemented without grassroots DHL training (56). Likewise, technically robust apps can be overshadowed by competing misinformation (57). Optimal digital health systems for pediatric care should integrate all three pillars: benchmarking during app development, embedding DHL education within community health worker (CHW) and caregiver training, and continuous social media monitoring to detect and respond to evolving narratives. This integration fosters iterative improvements, ensuring that technology aligns with real user needs (58).

Prioritizing participatory design enhances trust and user engagement, which is especially critical in pediatric health, where caregivers make key decisions. Involving communities in app design, content localization, and feedback mechanisms empowers users and fosters ownership. However, ethical challenges arise with digital tools, particularly concerning privacy in social media listening, which often involves analyzing publicly available but sensitive data sets. Benchmarking efforts must also focus on inclusiveness, ensuring that platforms are accessible to users with disabilities and those in remote areas. Furthermore, DHL education should avoid imposing top-down health behavior models and instead promote critical, context-aware decision-making that respects cultural nuances (59).

### Limitation

This mini-review is limited by its purposive scope, inclusion of only published English-language studies, and use of PubMed as the primary database with a targeted Web of Science supplement. Although adding Web of Science broadened interdisciplinary coverage, the search was not exhaustive and may still omit studies indexed in databases such as Scopus, Embase, IEEE Xplore, or regional indexes. No formal quality appraisal was conducted, limiting assessment of study rigor and comparability. Underrepresentation of gray literature and LMIC-specific evidence may also affect the completeness and generalizability of the framework. Social media data further raise ethical concerns regarding privacy, anonymization, and responsible handling of sensitive public content, alongside equity concerns related to the digital divide.

## Conclusion

The convergence of technology benchmarking, digital health literacy, and social media analysis provides a comprehensive framework for evaluating digital health interventions in pediatric care. Our mini-review of 15 studies, mapped to three pillars—technology benchmarking, digital health literacy, and social media listening—with a supporting cross-cutting evidence category highlights the multidimensional nature of digital health evaluation in low- and middle-income countries. Evidence from the technology benchmarking category underscores the importance of assessing infrastructure, regulatory approaches, and implementation barriers, while findings on digital health literacy emphasize the need for tools that are accessible, understandable, and equitable for diverse populations. Social media listening studies illustrate how real-time data can inform surveillance, communication, and engagement strategies, and cross-cutting studies provide typologies and frameworks that guide adoption and policy in resource-limited settings.
